# Dual-band high-Q quasi-BIC metasurface for refractive index sensing

**DOI:** 10.1515/nanoph-2025-0250

**Published:** 2025-09-05

**Authors:** Xunjie Lin, Yunfei Luo, Dongxian Li, Zhe Tang, Yue Li, Kaipeng Liu, Qingguo Du, Changtao Wang, Weisheng Yue

**Affiliations:** National Key Laboratory of Optical Field Manipulation Science and Technology, State Key Laboratory of Optical Technologies on Nano-Fabrication and Micro-Engineering, Institute of Optics and Electronics, Chinese Academy of Sciences, Chengdu 610209, China; School of Optoelectronics, University of Chinese Academy of Sciences, Beijing 100049, China; School of Optoelectronic Science and Engineering, University of Electronic Science and Technology of China, Chengdu 610054, China; School of Information Engineering, Wuhan University of Technology, 122 Luo Shi Road, Wuhan 430070, China

**Keywords:** BIC, all-dielectric metasurface, refractive index sensing

## Abstract

Sensitive and miniaturized optical sensing device is highly desirable in various biosensing applications. This study reports a dual-band, high-quality-factor (*Q* factor) quasi-bound states in the continuum (quasi-BIC) metasurface for refractive index sensing, operating across the visible (700–800 nm) and near-infrared (950–1,000 nm) spectral ranges. By incorporating asymmetric dual nanoholes into an all-dielectric silicon metasurface, symmetry-protected BIC modes are transformed into quasi-BIC, resulting in two distinct Fano-type resonance peaks. Numerical simulations and experimental validations demonstrate that precise control over resonance wavelengths and quality factors can be achieved by adjusting the nanohole radius and positional offsets (Δ), yielding a theoretical *Q*-factor of 2,250. The sensor exhibits a refractive index sensitivity of 151.6 nm/RIU for the visible band (Q-BIC I) and 61.1 nm/RIU for the near-infrared band (Q-BIC II), with a signal-to-noise ratio (SNR) of 285, significantly outperforming existing nanohole-based biosensors. Fabricated using CMOS-compatible processes, the device employs cost-effective visible-light detectors, eliminating the need for specialized infrared materials. This work advances the development of high-sensitivity, miniaturized refractive index sensing platforms, offering promising applications in biomedical diagnostics and environmental monitoring.

## 1 Introduction

Early diagnosis and fast tests of virus (such as COVID-19) has created high demand of easy-to-use, sensitive and rapid biochemical sensors. Refractive index (RI) sensing, which detects wavelength-shift of optical extinction peak caused by adsorption of molecules on surfaces of metallic nanostructures or films, has been extensively explored for detection of cancer molecules, influenza, as well as covid-19. Noble metallic nanostructures, such as nanosphere, nano-cubic, and nano-pyramids have been synthesized for RI sensing applications. Due to the flexibility in manipulating light at subwavelength scales [1], [2], [3], [4], metasurface has emerged as promising platform for RI sensing across diverse fields including environmental monitoring, food safety, chemistry, and biology [5], [6], [7], [8], [9], [10]. The adsorption of the target analyte molecules alters local dielectric environment, leading to a shift in metasufrace resonance peak. This enables rapid, label-free, and non-destructive detection of target samples [9], [11], [12]. High-Q resonances are usually preferred in refractive index sensing due to the improved sensitivity. In particular, asymmetric Fano resonances can yield extremely high quality factor, that is, sharp resonance peaks [13], [14], [15]. These sharp resonance peaks make it easier to observe shifts in the resonance position caused by changes in the refractive index, enabling metasurfaces to achieve high-sensitivity sensing functionality [16], [17]. At the same time, the linear relationship between resonance peak shifts and refractive index changes allows for the quantification of the target analyte’s characteristics [18]. While Fano resonance modes are often realized based on plasmonic sensors, their performance is often limited by the absorption losses in metal nanostructures [19].

To overcome these limitations, all-dielectric metasurfaces with quasi-bound states in the continuum has gained increasing attention in recent years [20], [21]. BICs are ideal states in which electromagnetic mode is fully confined within a finite region, theoretically resulting in infinite *Q*-factor. Under practical conditions, the symmetry breaking of the micro-nano structure leads to minor radiation losses, decoupling the BIC mode. At this point, the BIC mode transitions into a quasi-BIC, but still maintains sharp resonance peaks [22], [23]. The low-loss nature and excellent biocompatibility of all-dielectric metasurfaces expand their range of applications [24], [25], and the quasi-BIC mode ensures the performance of the metasurfaces, making them widely applied in optical sensing [26], [27], modulation [28], [29], and imaging [30], [31], [32], [33]. Quasi-BIC modes can be realized through various symmetry-breaking structures, such as asymmetrically inclined rectangular/elliptical nanocylinders [26], [27], crescent-shaped nanocylinders [34], asymmetric cylinder [35], [36], [37], and circular nanocylinders with different-shaped and positioned holes [32], [38], [39], [40], [41].

Metasurfaces can be designed to have specific resonant wavelengths depending on the application. In previous studies, quasi-BIC high-Q metasurfaces of Si were usually made to work at wavelengths above 1,000 nm [26], [27], [42], [43], [44]. This spectral range includes many characteristic molecular absorption fingerprints [45]. However, sensors operating at these wavelengths often require specialized detector materials, such as InGaAs (indium gallium arsenide) or InAs (indium arsenide). These components significantly increase the overall system cost, including detectors, light sources, filters, and other optical elements. Li et al. numerically demonstrated BICs working the visible light spectrum using GaP cuboids [48], which is compatible with visible light CMOS sensors. However, GaP is more expensive and its fabrication process is less mature than silicon. The 700–1,000 nm wavelength range, which falls within the optical response of common CMOS visible light sensors, offers an alternative to those infrared sensors. Although CMOS sensors have relative lower spectral response sensitivity in this wavelength range, the use of high-intensity light sources can compensate this limitation, improving the signal-to-noise ratio and maintaining accurate RI sensing performance, as the transmittance measurements are inherently relative.

Many studies have employed a single resonance peak for refractive index sensing. Recent studies have begun to introduce dual resonance peaks to reduce external interference from both the instrument and the environment, thereby enhancing the applicability and data reliability of refractive index sensing [46]. For example, Sun et al. theoretically demonstrated a high-Q, dual-band quasi-BIC metasurface operating in the 6–7 µm mid-infrared. The dual-band resonance design provides practical engineering benefits, including improved measurement accuracy through self-referenced drift compensation, and the potential for multi-analyte detection by selectively functionalizing the sensor based on two quasi-BIC modes [47], [48].

In comparison to other structures of cuboids or nanorods, nanohole arrays in a thin film represent a robust 2-D geometry, offering relatively fabricate simplicity via conventional semiconductor process. In addition, the nanohole regions are more accessible for solution analytes, facilitating efficient interaction between the analyte and the high-field region. In this work, we design and fabricate dual-nanohole all-dielectric metasurface sensor that supports high-Q quasi-BIC resonances. Through coordinated tuning of the nanohole radius and their relative positions, we break the symmetry to create two distinct quasi-BIC modes that can be independently controlled, as confirmed by both simulation and experiment. The resulting resonance peaks exhibit asymmetric Fano line shapes at two separate wavelengths: one in the visible light range (700–800 nm) and the other in the near-infrared range (950–1,000 nm). This study not only advances the miniaturization and practical implementation of high-sensitivity refractive index sensors but also introduces new strategies for multi-parameter optical sensing. Moreover, it provides a cost-effective solution for refractive index sensing based on CMOS technology, offering a promising path for future biosensing and environmental monitoring applications.

## 2 Asymmetric dual-nanohole sensor

**Figure 1:**
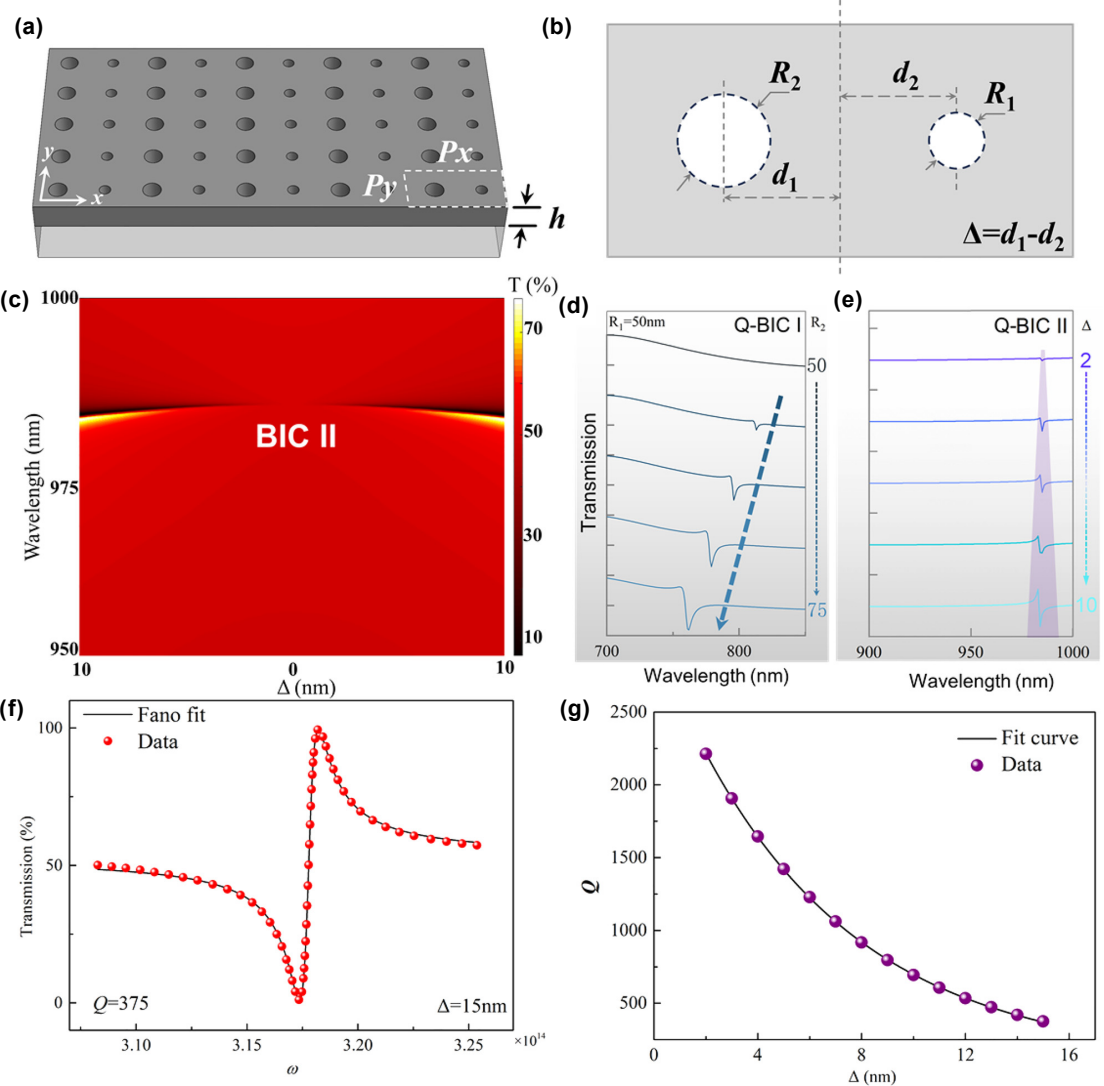
Asymmetric dual-nanohole metasurface refractive index sensor. (a) Top view. (b) Isometric view with dimension labels. Specific structural parameters: *Px* = 400 nm, *Py* = 200 nm, and *h* = 100 nm. (c) Simulated transmission spectra *T* for different values of Δ. (d) Simulated transmission spectra with different nanohole radius differences. (e) Simulated transmission spectra for different values of Δ. (f) Fano line shape fitting results (for Δ = 15 nm). (g) Variation trend of the *Q*-factor under different Δ conditions.

Figure 1(a) and (b) illustrate the proposed structure design with periodic asymmetric dual nanoholes, with 1a showing the top view and 1b presenting the isometric view. The nanoholes are located in the silicon layer, and the geometric parameters include the radius of the dual nanoholes (*R*_1_, *R*_2_), the positions of the nanoholes (*d*_1_, *d*_2_, Δ), the unit cell period (*Px*, *Py*), and the height of the silicon layer (*h*). The silicon was used because it is a widely used material in semiconductor industry and the fabrication process is mature. The substrate material is quartz. To determine the influence of the nanohole positions and radius on the metasurface’s resonance peaks and transmittance, numerical simulations were conducted using COMSOL Multiphysics. The light source used is the *y*-polarized light (perpendicular to the symmetry axis of the dual nanoholes), with periodic boundary conditions set in the *x* and *y* directions and a perfect matching layer in the *z* direction. The medium covered the metasurface is air.

Figure 1(c) displays the simulated transmission spectra *T* for different values of Δ, focusing on the near-infrared region (950–1,000 nm). The results show that when Δ = 0, the symmetry of the dual-nanohole positions is undisturbed, and no resonance peak is observed in the transmission spectrum. The variation of Δ excites the quasi-BIC resonance mode, as shown in Figure 1(e). As Δ increases, the depth and linewidth of the resonance peak increase gradually. The second symmetry-breaking method for this device is by modifying the radius difference of the dual nanoholes, Δ*R*. The quasi-BIC modes in the visible and near-infrared ranges are named Q-BIC I and Q-BIC II, respectively. These two quasi-BIC modes are independent of each other but both are forms of symmetry breaking. Figure 1(d) shows the variation in the transmission spectrum of quasi-BIC I when *R*_1_ = 50 nm and *R*_2_ increases from 50 nm to 75 nm, with the trend being similar to that of Q-BIC II.

Symmetry-protected BICs are typically associated with Fano resonances. Both Q-BIC I and Q-BIC II exhibit typical Fano line shapes in their resonance peaks. Therefore, the Fano formula shown in Equation (1) is used to fit the resonance peaks, where *ω*_0_ is the resonance frequency, and *a*, *b*, and *c* are constants. *γ* is related to the linewidth of the resonance peak, and the quality factor *Q* is given by *Q* = *ω*_0_/2*γ* [26], [39].

(1) $$T_{\text{Fano}}\left(\omega \right)=\left|a+jb+\frac{c}{\omega -\omega_{0}+j\gamma }\right|$$

Figure 1(f) presents the Fano line shape fitting when Δ = 15 nm. The calculated *Q* factor of the Q-BIC I resonance peak is relatively low, below 200. Therefore, a detailed simulation of the Q-BIC II resonance mode was carried out in this study. Figure 1(g) illustrates the trend of the quality factor *Q* for different values of Δ. As the linewidth narrows, the quality factor of the resonance peak increases gradually. When Δ = 2 nm, the quality factor reaches 2,250. However, as shown in Figure 1(d) and (e), the narrowing of the linewidth is accompanied by a decrease in the depth of the resonance. Therefore, to ensure the feasibility of the experiment, it is necessary to impose an appropriate limitation on the quality factor.

To further understand the resonance characteristics of the Q-BIC I and Q-BIC II modes, we computed the near-field electromagnetic distributions of the asymmetric dual-nanohole metasurface at the resonance wavelengths (with parameters: *R*_1_ = 80 nm, *R*_2_ = 50 nm, and Δ = 10 nm). Based on the Cartesian coordinate system, we evaluated the multipole moments including the electric dipole (ED), magnetic dipole (MD), toroidal dipole (TD), electric quadrupole (EQ), and magnetic quadrupole (MQ), which are defined as follows [46], [49]:

(2) $$\text{ED}:P=\frac{1}{i\omega }\int \vec{j}d^{3}r$$

(3) $$\text{MD}:M=\frac{1}{2c}\int \left(r\times j\right)d^{3}r$$

(4) $$\text{TD}:T=\frac{1}{10c}\int \left[\left(r\cdot j\right)r-2r^{2}j\right]d^{3}r$$

(5) $$\text{EQ:}Q_{\alpha \beta }^{\left(e\right)}=\frac{1}{2i\omega }\int \left[r_{\alpha }j_{\beta }+r_{\beta }j_{\alpha }-\frac{2}{3}\left(r\cdot j\right)\delta_{\alpha ,\beta }\right]d^{3}r$$

(6) $$\text{MQ:}Q_{\alpha \beta }^{\left(m\right)}=\frac{1}{3c}\int \left[{\left(r\times j\right)}_{\alpha }r_{\beta }+{\left(r\times j\right)}_{\beta }r_{\alpha }\right]d^{3}r$$

where *c* is the speed of light, *ω* is the angular frequency, **r** represents the position vector, **j** is the displacement current density and *δ*_*α*,*β*_ denotes the Kronecker delta. Based on the multipole decomposition results, the total scattered power *I* corresponding to different dipoles can be calculated using the following formulas [50]:

(7) $$\begin{array}{ll} I & =\frac{2\omega^{4}}{3c^{3}}{\left|P\right|}^{2}+\frac{2\omega^{4}}{3c^{3}}{\left|M\right|}^{2}+\frac{\omega^{6}}{5c^{5}}{\left|Q_{\alpha \beta }^{\left(e\right)}\right|}^{2}\\ & \;+\frac{\omega^{6}}{20c^{5}}{\left|Q_{\alpha \beta }^{\left(m\right)}\right|}^{2}+\frac{2\omega^{6}}{3c^{5}}{\left|T\right|}^{2} \end{array}$$

**Figure 2:**
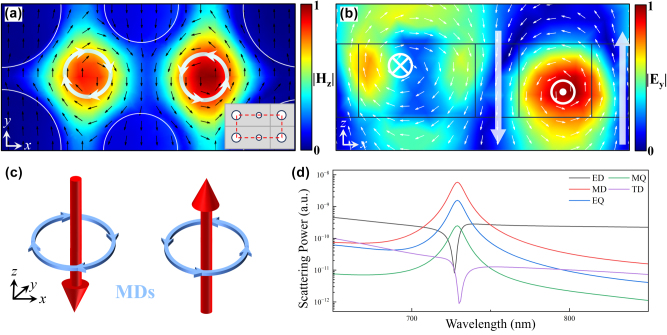
Resonance characteristics of Q-BIC I. (a) Magnetic field distribution in the *x*–*y* plane at the resonance wavelength. (b) Electric field distribution in the *x*–*z* plane at the resonance wavelength. (c) Schematic of two pairs of magnetic dipole sources. (d) Contribution of different multipole excitations.

**Figure 3:**
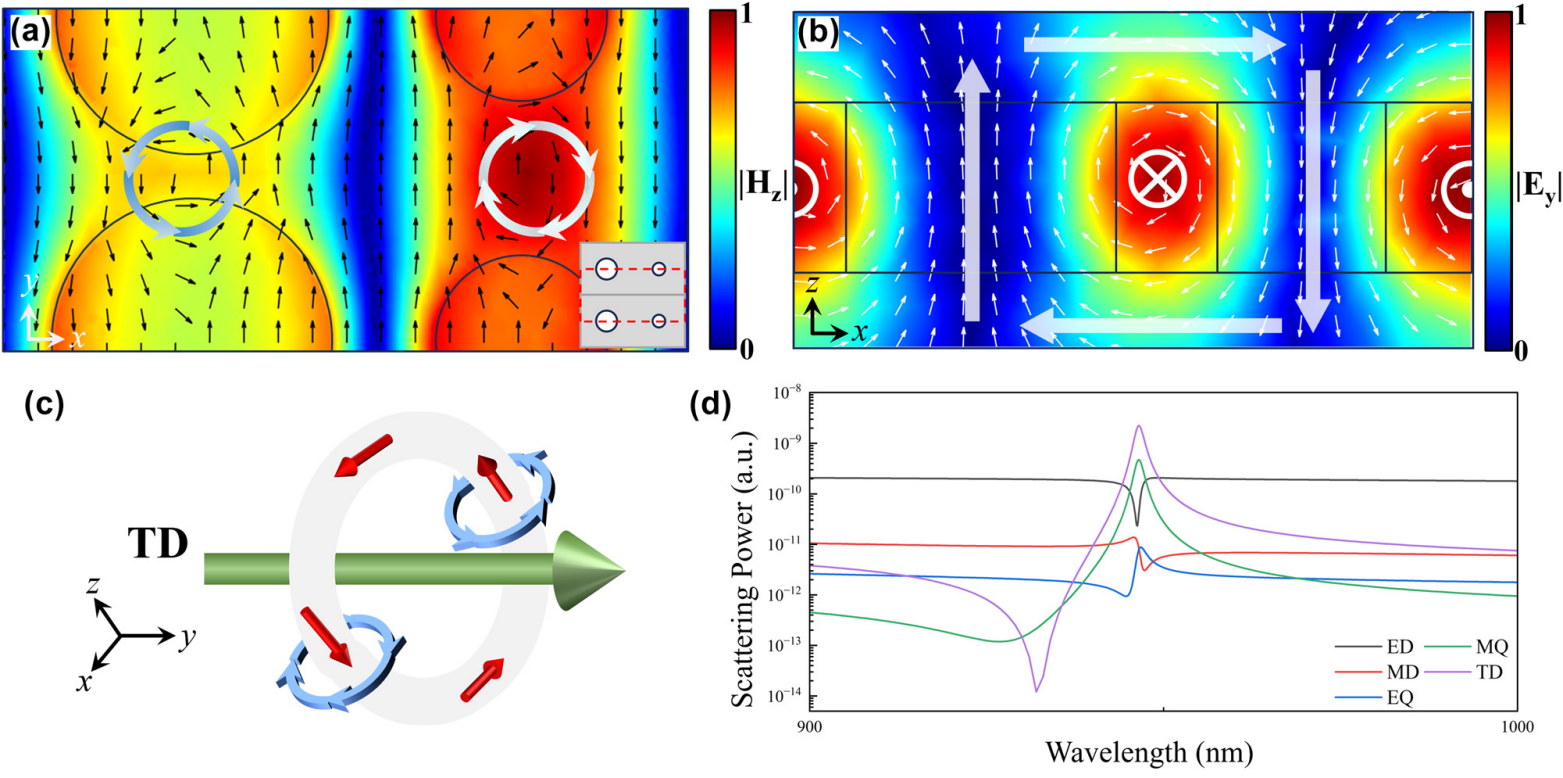
Resonance characteristics of Q-BIC II. (a) Magnetic field distribution in the *x*–*y* plane at the resonance wavelength. (b) Electric field distribution in the *x*–*z* plane at the resonance wavelength. (c) Schematic of toroidal dipole source. (d) Contribution of different multipole excitations.

Figures 2 and 3 present the electromagnetic field distributions, schematic diagrams of electromagnetic sources, and the corresponding multipolar scattering powers at the resonance wavelengths of Q-BIC I and Q-BIC II. In the electromagnetic field distribution diagrams, the magnitudes of the electromagnetic fields have been normalized. The black and white arrows indicate the instantaneous directions of the electric and magnetic fields, respectively. The enhanced fields are mainly concentrated in the inter-element regions. Therefore, the magnetic field distribution in the *x*–*y* plane has been appropriately adjusted to fully display the complete vortex structures, as illustrated in the insets of Figures 2(a) and 3(a).

As shown in Figure 2(d), the Q-BIC I resonance is dominated by MD. Figure 2(a) shows the electric field vector distribution in the *x*–*y* plane at the Q-BIC I resonance wavelength (*λ*_1_ = 731 nm), where two electric field vortices are observed near the gap between the dual-nanohole and the unit cell boundary. In Figure 2(b), the white arrows denote the magnetic field vectors in the *x*–*z* plane at *y* = −100 nm. Combined with the displacement current distribution in Figure 2(a), these counter-rotating current loops give rise to two *z*-directed MDs. Figure 2(c) shows a schematic of two representative MD source pairs, corresponding to the two current loops in Figure 2(a). Although Figure 2(b) exhibits characteristics of a TD mode, the presence of two oppositely oriented TDs within the unit cell leads to destructive coupling, thus reducing the overall TD scattering contribution. Consequently, the Q-BIC I resonance mainly arises from the combined contributions of MD mode.

Figure 3(a) displays the electric field vector distribution in the *x*–*y* plane at the Q-BIC II resonance wavelength (*λ*_2_ = 947 nm). Similar to Q-BIC I, multiple electric field vortices are observed. However, the vortex positions shift from the gap region to the edges of the nanoholes. As shown in Figure 3(b), counter-rotating current loops at the edges of the dual-nanohole generate two oppositely directed MDs, which collectively form a MQ. Meanwhile, a closed magnetic field loop is observed in the *x*–*z* plane, as shown in Figure 3(c), indicating the presence of a TD mode. These electromagnetic field distributions are consistent with the multipole decomposition results in Figure 2(d), confirming that the Q-BIC II resonance is mainly dominated by the excitation of TD mode.

Electron-beam lithography was used to fabricate the asymmetric dual-nanohole metasurfaces on quartz glass. The sensor dimensions were determined based on the optimal simulation results obtained from COMSOL parameter sweeps [51]. Since the performance of quasi-BIC devices is highly sensitive to structural dimensions, we took both fabrication conditions and the *Q*-factor into consideration. As a result, we selected the following parameters for fabrication: *Px* = 400 nm, *Py* = 200 nm, *R*_1_ = 50 nm, and *R*_2_ = 80 nm. The nanohole array has area of 200 µm × 200 µm. The patterned areas are labelled with letters A–C for differentiation. After fabrication, the metasurface was characterized using a scanning electron microscope (SEM) to assess its morphology and measure fabrication tolerances. A set of nanohole structures with good fabrication quality was selected to modify the COMSOL simulation model. The design dimensions and the measured fabrication dimensions are shown in Table 1.

**Table 1:** Design and fabricated dimensions of the asymmetric dual-nanohole metasurface.

	Design dimensions			Manufactured dimensions		
	*R*_1_ (nm)	*R*_2_ (nm)	Δ (nm)	*R*_1_ (nm)	*R*_2_ (nm)	Δ (nm)
A	60	80	0	62	82	0
B			5	62	88	0
C			10	62	88	8

**Figure 4:**
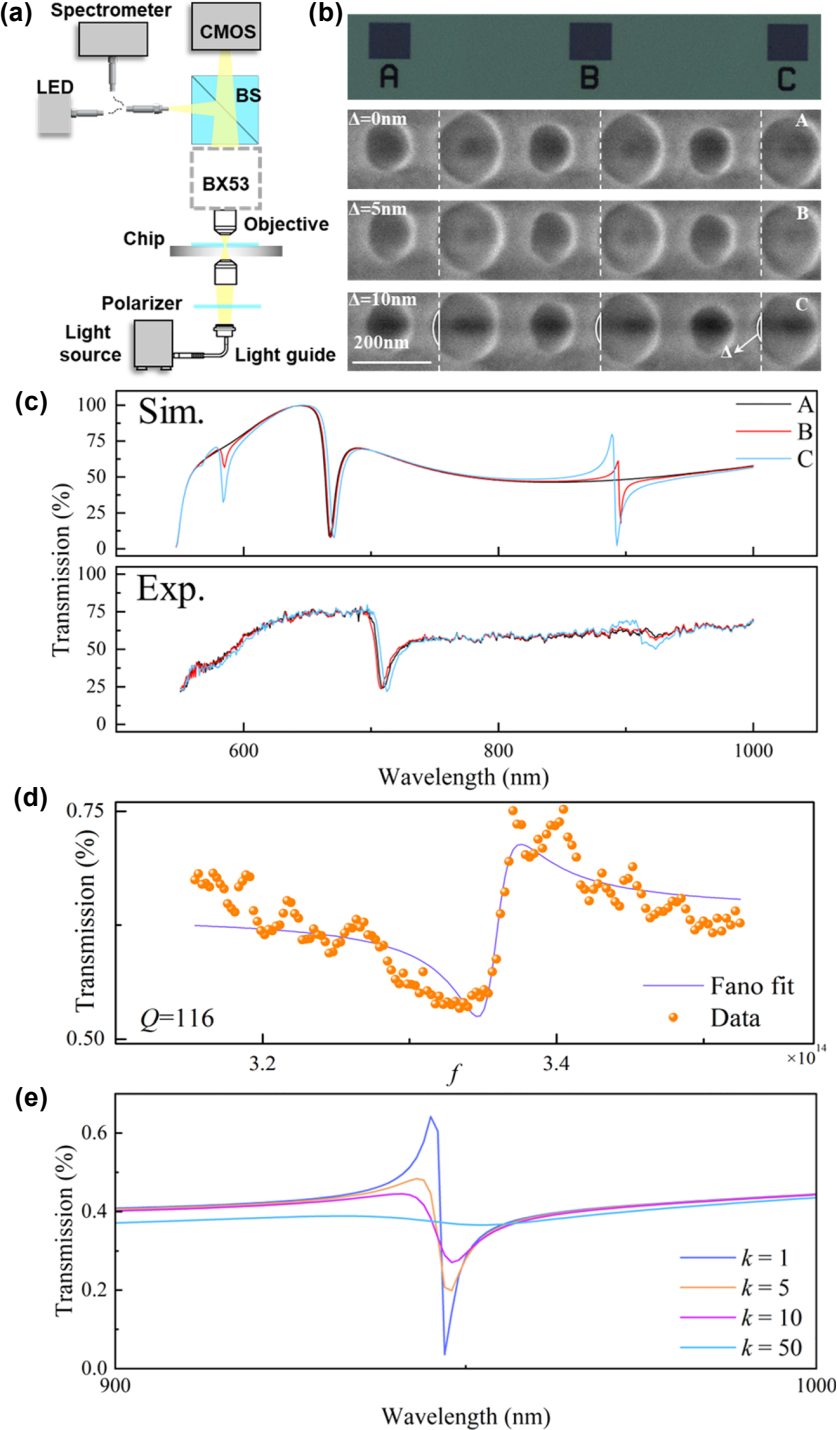
Micro-region spectral testing system for the asymmetric dual-nanohole metasurface. (a) Schematic of the testing system. (b) Bright-field microscope image and SEM image of the BIC array. (c) Comparison of simulated and experimental transmission spectra of the BIC array. (d) Fano fitting results of the resonance peak for sample C. (e) The simulated transmission spectra under different extinction coefficients of Si.

The optical transmittance was measured with laboratory-made microspectrometer, which is shown in Figure 4(a). The microspectrometer is based on an optical microscope (Olympus BX53), with a *Y*-shaped optical fiber used to define the micro-area and collect spectral information. The spectrometer used has a wavelength range of 350–1,100 nm and a wavelength resolution of 0.8 nm. Figure 4(b) displays the bright-field microscope image of the BIC array and the SEM image. The SEM image is aligned along the edge of the circular holes to display the Δ variation. A comparison between the nanohole edges of regions A and C reveals differences in their positions. This result matches the fabrication dimensions measured in Table 1, which may be attributed to exposure and etching processes causing discrepancies between the expected and actual nanohole dimensions and positions.

As shown in Figure 4(c), we measured the transmission spectrum of the BIC array and modified the COMSOL model based on the SEM image to obtain the simulated transmittance curve. The resonance peak of region C was fitted using the Fano model, with the fitting result shown in Figure 4(d). The quality factor obtained from the fit is 116, while the simulated *Q* factor is 693. The difference in the depth may be attributed to energy losses caused by surface roughness and defects in the dielectric layer [52]. Figure 4(e) shows the simulated transmission spectra at different multiples of the extinction coefficient *k*. It can be observed that as *k* increases, the depth of the Q-BIC II resonance decreases, which is consistent with the experimental observation of a reduced *Q* factor.

**Figure 5:**
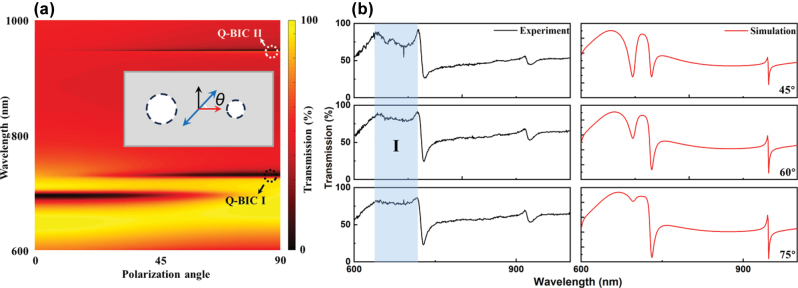
Polarization-dependent transmission spectra of the metasurface. (a) Pseudocolor map of transmission spectra as a function of polarization angle, with the inset illustrating the polarization orientation. Specific structural parameters: *Px* = 400 nm, *Py* = 200 nm, *R*_1_ = 50 nm, *R*_2_ = 80 nm, Δ = 10 nm and *h* = 100 nm. (b) Comparison of experimental and simulated transmission spectra at polarization angles of 45°, 60°, and 75°.

We further simulated the transmission spectra of the sensor under polarization angles ranging from 0° to 90°. As shown in Figure 5(a), as *θ* decreases from 90° to 0°, the Q-BIC I and Q-BIC II resonance modes gradually disappear. Meanwhile, a new symmetric Lorentz-type resonance peak emerges near 700 nm. This new resonance coexists with the Q-BIC I and II resonances under excitation conditions where the incident electric field contains both *x*- and *y*-polarized components. Figure 5(b) presents a comparison between experimental and simulated transmission spectra at three polarization angles (45°, 60°, and 75°). The simulation results show that as the polarization angle decreases, the new resonance peak becomes increasingly clearer, while the peak-to-valley contrast of the Q-BIC I and II resonances gradually decreases. In region I of Figure 5(b), no distinct resonance peak is observed in the experimental results. But the variation in transmittance between the peak and valley in this region matches well with the simulation. Overall, the experimental and simulated results exhibit consistent trends, confirming that the polarization angle plays a critical role in modulating the resonance modes.

To verify the variation of Q-BIC I, specifically the impact of the radius difference Δ*R* of the dual nanoholes on the quasi-BIC resonance peak, we redesigned four sets of BIC metasurfaces. The fabrication process accounted for manufacturing errors, and the design dimensions and the resulting fabrication dimensions are shown in Table 2.

**Table 2:** Design and fabricated dimensions of the asymmetric dual-nanohole metasurface.

	Design dimensions			Manufactured dimensions		
	*R*_1_ (nm)	*R*_2_ (nm)	Δ (nm)	*R*_1_ (nm)	*R*_2_ (nm)	Δ (nm)
A	50	70	0	50	70	0
B		75		52	76	
C		80		52	80	
D	60	80		59	82	

**Figure 6:**
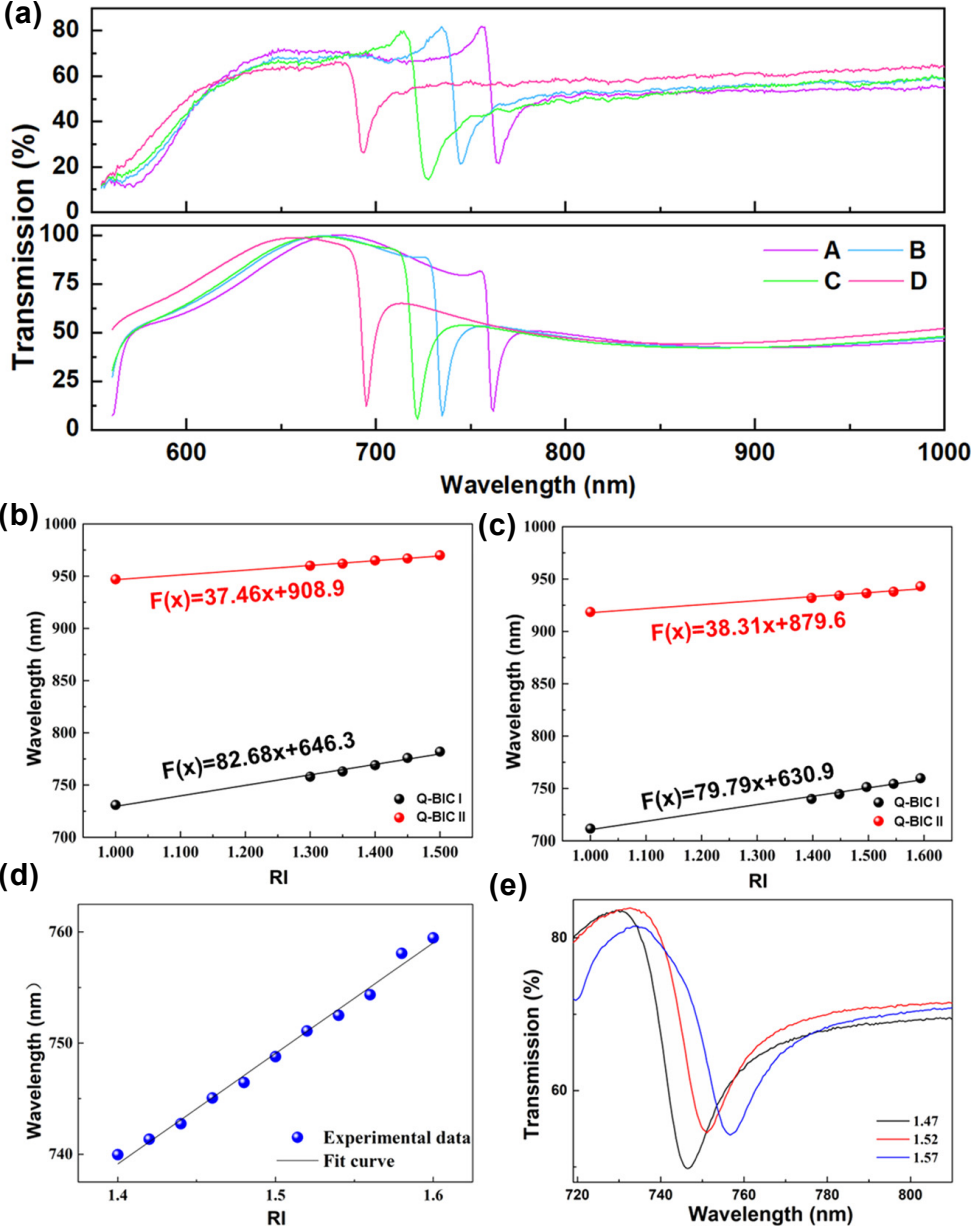
Refractive index sensing performance of the asymmetric dual-nanohole metasurface. (a) Effect of the radius difference of the dual-nanohole on the BIC resonance peak. (b) Simulated and (c), (d) experimental transmission spectra curves of standards with different refractive indices. (e) Experimental validation results.

Figure 6(a) displays a comparison of the transmission spectra and the fitted curves for arrays with different nanohole radius combinations (A–D). The resonance wavelengths obtained from both simulations and experiments are consistent. The experimental Fano lineshapes show more pronounced short-wave features. For regions A–C, as the radius of *R*_2_ increases, the resonance peak shifts toward the short-wavelength direction, in agreement with the trend shown in Figure 1(d). However, the asymmetry of the Fano lineshape appears to remain relatively unchanged due to the larger size of *R*_2_.

An important indicator of refractive index sensors is the sensitivity *S*, which is defined as the ratio of wavelength change to refractive index change. We tested the refractive index sensing performance using standard refractive index solutions, selecting different reference materials with refractive indices ranging from 1.4 to 1.6, specifically: 1.3978, 1.4477, 1.4967, 1.5453, and 1.5937. The corresponding simulated and experimental transmission spectra are presented in Figure 6(b) and (c), respectively. As the refractive index increases, a redshift in the resonance wavelength is observed. The experimentally measured sensitivities are 79.79 nm/RIU for Q-BIC I and 38.31 nm/RIU for Q-BIC II, with relative errors of 2.27 % and 3.50 % compared to the simulation results. Meanwhile, we further performed a calibration experiment using standard refractive index liquids (Cargill, USA) with an interval of 0.02. The calibration results are shown in Figure 6(d). To evaluate the sensor’s performance, three standard solutions with refractive indices of 1.47, 1.52, and 1.57 were selected for testing. Based on the shifts in resonance wavelength, the measured relative errors were 0.249 %, 0.412 %, and 0.024 %, respectively. These results demonstrate that the sensor exhibits reasonable stability and accuracy for practical RI sensing applications. For this quasi-BIC refractive index sensors, the enhanced sensitivity is fundamentally tied to the unique modal properties of the structure. The quasi-BIC states concentrate the electric field either inside the nanoholes or in the inter-element regions, enhancing the coupling between the optical mode and the analyte. In addition to this strong field–matter interaction, the suppression of radiative loss inherent to quasi-BIC modes yields ultra-narrow linewidths, allowing even slight refractive index changes to produce significant resonance shifts. Donato et al. proposed that the performance of biosensors should also consider the signal-to-noise ratio (SNR) of the resonance curve [32]. The definition of SNR is given by Equation (8).

(8) $$\text{SNR}=\left(R_{\max}-R_{\min}\right)/\sigma_{\text{spectrum}}$$

where *R*_max_ − *R*_min_ is the resonance amplitude, i.e., the peak-to-valley difference, and *σ*_spectrum_ is the standard deviation of the signal noise. After calculation, the SNR of Q-BIC I was found to be 285, significantly higher than the SNR of 160 reported for nanohole structures in the literature [32]. Meanwhile, integration with artificial intelligence techniques may further enhance the sensor’s robustness and adaptability in complex or noisy environments [53].

## 3 Conclusions

In this work, we present a refractive index sensor based on a quasi-BIC asymmetric dual-nanohole metasurface. By adjusting the radius and positions of the nanoholes, dual resonance peaks are achieved within the operating wavelength range of commercial color CMOS sensors. The sensor exhibits a high theoretical *Q* factor of up to 2,250 and a refractive index sensing sensitivity of 79.79 nm/RIU. Moreover, the sensor demonstrates a SNR of 285, calculated from the resonance amplitude and noise standard deviation, significantly surpass the SNR of 160 reported for conventional nanohole-based biosensors. These results highlight strong potential of asymmetric dual-nanohole metasurface sensors as a high-sensitivity platform for refractive index sensing, offering promising opportunities for compact, cost-effective optical sensing and imaging applications in areas of biosensing and environment monitoring.

## 4 Materials and methods

### 4.1 Numerical simulations

This study uses COMSOL Multiphysics (version 6.2) for simulating and calculating the transmission spectrum of the proposed asymmetric dual-nanohole metasurface. The base and dielectric layer are modeled as rectangular prisms, with unit cell dimensions of *Px* = 400 nm and *Py* = 200 nm. The dielectric layer has a thickness of 100 nm. The dimensions of the dual nanoholes within the dielectric layer are parametrically scanned starting from 50 nm. One of the nanoholes has a fixed diameter of d1 = 100 nm, while the other nanohole’s diameter (d2) is varied between 80 nm and 120 nm to investigate the impact of Q-BIC II on the resonance peaks. The mesh element size within the model is controlled by the physical fields.

### 4.2 Fabrication

The substrate of the asymmetric dual-nanohole metasurface is made of 0.7 mm thick quartz glass. A 100 nm thick silicon dielectric layer is deposited onto the substrate using chemical vapor deposition (CVD). Photoresist is then spin-coated onto the dielectric layer, followed by electron beam lithography (EBL) to pattern the designed asymmetric dual-nanohole structure onto the photoresist. After development, a 10 nm thick chromium layer is deposited on the sample surface. The micro-nano structure is fabricated using inductively coupled plasma (ICP) dry etching. Finally, the chromium layer is removed using a chromium etchant, completing the sensor fabrication process.

### 4.3 Optical measurement setup

The optical measurement setup is constructed around an Olympus BX53 microscope, with white light provided by a halogen lamp. The light is transmitted through a light guide, passing through a linear polarizer and a microscope objective into the system. The transmittance data and image signals are directed through a 20× objective (NA = 0.4) for near-infrared (Mitutoyo, Plan Apo NIR) and transmitted to a 5:5 beam splitter. The two resulting signals are separately collected by a CMOS camera (MER2-2000-19U3C) and a spectrometer (spectral range: 350–1,100 nm, resolution: 0.8 nm). The spectrometer and an LED are connected to the system via a *Y*-type optical fiber, with the fiber position adjustable using a coaxial mechanical setup, and a lens placed behind the fiber to optimize coupling efficiency. The LED is used to define the micro-area corresponding to the transmittance test.

## References

[1] Pu M. (2015). Catenary optics for achromatic generation of perfect optical angular momentum. *Sci. Adv.*.

[2] Xie X. (2021). Generalized Pancharatnam-Berry phase in rotationally symmetric meta-atoms. *Phys. Rev. Lett.*.

[3] Guo Y. (2021). Spin-decoupled metasurface for simultaneous detection of spin and orbital angular momenta via momentum transformation. *Light: Sci. Appl.*.

[4] Guo Y. (2022). Classical and generalized geometric phase in electromagnetic metasurfaces. *Photonics Ins.*.

[5] Xu Y. (2019). Optical refractive index sensors with plasmonic and photonic structures: promising and inconvenient truth. *Adv. Opt. Mater.*.

[6] Li H., Kim J. T., Kim J.-S., Choi D.-Y., Lee S.-S. (2023). Metasurface-incorporated optofluidic refractive index sensing for identification of liquid chemicals through vision intelligence. *ACS Photonics*.

[7] Zhao Y. (2024). Detection of food additives based on an integrated self-injected metasurface microfluidic sensor. *Opt. Express*.

[8] Tabassum S., Nayemuzzaman S., Kala M., Kumar Mishra A., Mishra S. K. (2022). Metasurfaces for sensing applications: gas, bio and chemical. *Sensors*.

[9] Ali Khan S. (2022). Optical sensing by metamaterials and metasurfaces: from physics to biomolecule detection. *Adv. Opt. Mater.*.

[10] Maleki J., Fathi D. (2024). Advanced terahertz refractive index sensor in all-dielectric metasurface utilizing second order toroidal quasi-BIC modes for biochemical environments. *Sci. Rep*..

[11] Shen Y. (2013). Plasmonic gold mushroom arrays with refractive index sensing figures of merit approaching the theoretical limit. *Nat. Commun*..

[12] Qin J. (2022). Metasurface micro/nano-optical sensors: principles and applications. *ACS Nano*.

[13] Miroshnichenko A. E., Flach S., Kivshar Y. S. (2010). Fano resonances in nanoscale structures. *Rev. Mod. Phys.*.

[14] Luk’yanchuk B. (2010). The Fano resonance in plasmonic nanostructures and metamaterials. *Nat. Mater.*.

[15] Limonov M. F., Rybin M. V., Poddubny A. N., Kivshar Y. S. (2017). Fano resonances in photonics. *Nat. Photonics*.

[16] Zafar R., Salim M. (2015). Enhanced figure of merit in Fano resonance-based plasmonic refractive index sensor. *IEEE Sens. J.*.

[17] Zhang Y. (2018). High-quality-factor multiple Fano resonances for refractive index sensing. *Opt. Lett.*.

[18] Liang C., Lai J., Lou S., Duan H., Hu Y. (2022). Resonant metasurfaces for spectroscopic detection: physics and biomedical applications. *Adv. Devices Instrum.*.

[19] Adhikari R., Chauhan D., Mola G. T., Dwivedia R. P. (2024). A review of the current state-of-the-art in Fano resonance-based plasmonic metal-insulator-metal waveguides for sensing applications. *Opto-Electron. Rev.*.

[20] Hsu C. W., Zhen B., Stone A. D., Joannopoulos J. D., Soljačić M. (2016). Bound states in the continuum. *Nat. Rev. Mater.*.

[21] Kang M., Liu T., Chan C. T., Xiao M. (2023). Applications of bound states in the continuum in photonics. *Nat. Rev. Phys.*.

[22] Plotnik Y. (2011). Experimental observation of optical bound states in the continuum. *Phys. Rev. Lett.*.

[23] Yao J. (2023). Bound states in continuum in periodic optical systems. *Chin. Opt.*.

[24] Ako R. T., Upadhyay A., Withayachumnankul W., Bhaskaran M., Sriram S. (2020). Dielectrics for terahertz metasurfaces: material selection and fabrication techniques. *Adv. Opt. Mater.*.

[25] Tian J., Li Q., Belov P. A., Sinha R. K., Qian W., Qiu M. (2020). High-Q all-dielectric metasurface: super and suppressed optical absorption. *ACS Photonics*.

[26] Liu Z. (2023). Phase interrogation sensor based on all-dielectric BIC metasurface. *Nano Lett.*.

[27] Chen W., Li M., Zhang W., Chen Y. (2023). Dual-resonance sensing for environmental refractive index based on quasi-BIC states in all-dielectric metasurface. *Nanophotonics*.

[28] CaiWei Tan T. (2021). Active control of nanodielectric-induced THz quasi-BIC in flexible metasurfaces: a platform for modulation and sensing. *Adv. Mater.*.

[29] Hu T., Qin Z., Chen H., Chen Z., Xu F., Wang Z. (2022). High-Q filtering and dynamic modulation in all-dielectric metasurfaces induced by quasi-BIC. *Opt. Express*.

[30] Zhou C., Qu X., Xiao S., Fan M. (2020). Imaging through a Fano-resonant dielectric metasurface governed by quasi-bound states in the continuum. *Phys. Rev. Appl.*.

[31] Saadatmand S. B., Ahmadi V., Hamidi S. M. (2023). Quasi-BIC based all-dielectric metasurfaces for ultra-sensitive refractive index and temperature sensing. *Sci. Rep*..

[32] Conteduca D., Barth I., Pitruzzello G., Reardon C. P., Martins E. R., Krauss T. F. (2021). Dielectric nanohole array metasurface for high-resolution near-field sensing and imaging. *Nat. Commun*..

[33] Wang J., Maier S. A., Tittl A. (2022). Trends in nanophotonics-enabled optofluidic biosensors. *Adv. Opt. Mater.*.

[34] Wang J., Kühne J., Karamanos T., Rockstuhl C., Maier S. A., Tittl A. (2021). All-dielectric crescent metasurface sensor driven by bound states in the continuum. *Adv. Funct. Mater.*.

[35] Wang Y., Han Z., Du Y., Qin J. (2021). Ultrasensitive terahertz sensing with high-Q toroidal dipole resonance governed by bound states in the continuum in all-dielectric metasurface. *Nanophotonics*.

[36] Chen Y., Li Y., Hu Z., Wang Z., Li Z., Wang J. (2023). High-performance quality factor based sensor with diagonal cylinder metasurface of the bound state in the continuum. *Photonic Sens.*.

[37] Liu N., Wang S., Lv J., Zhang J. (2023). Achiral nanoparticle trapping and chiral nanoparticle separating with quasi-BIC metasurface. *Opt. Express*.

[38] Mi Q. (2021). High-quality-factor dual-band Fano resonances induced by dual bound states in the continuum using a planar nanohole slab. *Nanoscale Res. Lett.*.

[39] Du X., Xiong L., Zhao X., Chen S., Shi J., Li G. (2022). Dual-band bound states in the continuum based on hybridization of surface lattice resonances. *Nanophotonics*.

[40] Zheng Z. (2023). Third-harmonic generation and imaging with resonant Si membrane metasurface. *Opto-Electron. Adv.*.

[41] Fan J., Xue Z., Wang H., Cong L. (2023). Hybrid bound states in the continuum in terahertz metasurfaces. *2023 Cross Strait Radio Science and Wireless Technology Conference (CSRSWTC)*.

[42] Jahani Y. (2021). Imaging-based spectrometer-less optofluidic biosensors based on dielectric metasurfaces for detecting extracellular vesicles. *Nat. Commun*..

[43] Watanabe K., Iwanaga M. (2023). Nanogap enhancement of the refractometric sensitivity at quasi-bound states in the continuum in all-dielectric metasurfaces. *Nanophotonics*.

[44] Yesilkoy F. (2019). Ultrasensitive hyperspectral imaging and biodetection enabled by dielectric metasurfaces. *Nat. Photonics*.

[45] Wei Y. (2023). A mid-IR tunable graphene metasurface for ultrasensitive molecular fingerprint retrieval and refractive index sensing. *J. Mater. Chem. C*.

[46] Wang T. (2024). Dual high-Q Fano resonances metasurfaces excited by asymmetric dielectric rods for refractive index sensing. *Nanophotonics*.

[47] Sun W. (2024). Potential of high Q dual band mid-infrared metasurfaces with quasi-BIC for refractive index sensing. *Opt. Laser Technol.*.

[48] Li Z. (2023). Optical sensing and switching in the visible light spectrum based on the bound states in the continuum formed in GaP metasurfaces. *Appl. Surf. Sci.*.

[49] Yang L., Yu S., Li H., Zhao T. (2021). Multiple Fano resonances excitation on all-dielectric nanohole arrays metasurfaces. *Opt. Express*.

[50] Li N. (2025). Ultrasensitive metasurface sensor based on quasi-bound states in the continuum. *Nanophotonics*.

[51] Wu X. (2024). Exciton polariton condensation from bound states in the continuum at room temperature. *Nat. Commun.*.

[52] Hsiao H., Hsu Y., Liu A., Hsieh J., Lin Y. (2022). Ultrasensitive refractive index sensing based on the quasi-bound states in the continuum of all-dielectric metasurfaces. *Adv. Opt. Mater.*.

[53] Badloe T. (2024). Artificial intelligence-enhanced metasurfaces for instantaneous measurements of dispersive refractive index. *Adv. Sci.*.

